# The complete genome sequence of elite bread wheat cultivar, “Sonmez”

**DOI:** 10.12688/f1000research.121637.1

**Published:** 2022-06-06

**Authors:** Bala Ani Akpinar, Philippe Leroy, Nathan Watson-Haigh, Ute Baumann, Valerie Barbe, Hikmet Budak

**Affiliations:** 1 Genomics and Genome Editing, Montana BioAg. Inc, Missoula, Montana, 59802, USA; 2 Diversité et Écophysiologie des Céréales, INRAE-UCA, UMR 1095, Génétique,, Clermont-Ferrand, 10951095, France; 3 School of Agriculture, Food and Wine, University of Adelaide, Plant Genomics Centre, Waite Campus, Hartley Grove, Urrbrae SA 5064, Australia, Adelaide, Australia; 4 South Australian Genomics Centre, SAHMRI, North Terrace, Adelaide SA 5000, Australia (NSWH), Adelaide, Australia; 5 Australian Genome Research Facility, Victorian Comprehensive Cancer Centre, Melbourne, VIC 3000, Australia; 6 Génomique Métabolique, Genoscope, Institut François Jacob, CEA, CNRS, Evry, France

**Keywords:** Wheat, genome sequencing, Triticum aestivum, yield, Sonmez

## Abstract

High-yielding crop varieties will become critical in meeting the future food demand in the face of worsening weather extremes and threatening biotic stressors. The bread wheat cultivar Sonmez-2001 is a registered variety that is notable for its performance under low-irrigation conditions, which further improves upon irrigation. Additionally, Sonmez-2001 is resilient against certain biotic stressors, particularly soil-borne pathogens.

Here, we provide a reference-guided whole genome sequence of Sonmez-2001, assembled into 21 chromosomes of the A, B and D genomes and totaling 13.3 gigabase-pairs in length. Additionally, a *de novo* assembly of an additional 1.05 gigabase-pairs was generated that represents either Sonmez-specific sequences or sequences that considerably diverged between Sonmez and Chinese Spring. Within this *de novo* assembly, we identified 35 gene models, of which 11 were high-confidence, that may contribute to the favorable traits of this high-performing variety. We identified up to 24 million sequence variants, of which up to 2.4% reside in coding sequences, that can be used to develop molecular markers that should be of immediate use to the cereal community.

## Introduction

*Triticum aestivum* cv. Sonmez-2001 (Sonmez, hereafter) is a registered, elite bread wheat variety that has been bred particularly for drylands. Accordingly, Sonmez exhibits remarkable tolerance against drought and performs considerably better than its ancestor, Bezostaya-1, in terms of yield, stress tolerance and disease resistance. Sonmez variety is notable for high yield and grain quality, building up to ≈15% protein content, under rain-fed conditions, both of which further improve with supplemental irrigation. Sonmez is also highly resistant against causal agents of devastating diseases, in particular, cereal cyst nematode and yellow rust. Sonmez has superior resistance against soil-borne pathogens and exhibit good tolerance against diseases affecting leaves and inflorescence. Due to these attributes, Sonmez is the cultivar of choice for most of the Central Anatolian Plateau. Facing a fast-growing world population, estimated to reach over 9 billion people in the next three decades, and changing climate trends with destructive effects on agriculture, securing the food demand of upcoming generations will require extensive improvements in crop yields. With cereals being the staple food for the developing world, Sonmez is a promising candidate that can contribute to meeting this demand. Here, we report a reference-guided sequence of the Sonmez genome, and its comparative analysis with the reference species, *Triticum aestivum* genotype Chinese Spring, for which extensive data, including a high-quality genome sequence, is available.

## Methods

A paired-end (PE) library with an insert size of 350 base-pair was produced and sequenced on Illumina HiSeq 4000 platform at Genoscope, National Center of Sequencing, (Évry-Courcouronnes, France), generating almost 3.3 billion 2×150 bp reads. The 970.6 gigabase-pair (Gbp) of PE reads passing quality filters were mapped against the *T. aestivum* Chinese Spring (CS) RefSeq v1.0 genome ^1^ in a two-step approach. In the first step, an ungapped alignment was performed using BioKanga v3.4.5 using default parameters but allowing for two mismatches per 100 bp (--substitutions=2). In the second step, the unmapped reads were mapped with Bowtie2 v2.3.0, ^2^ allowing a single insertion/deletion of length ≤ 9 bp with zero mismatches (--very-sensitive --ignore-quals --mp 999,999 --np 999 --rdg 10,1 --rfg 10,1 --score-min L,-19,0 --n-ceil L,0,0). Read alignments from both mapping steps were merged using Sambamba v0.6.5. ^3^ Regions containing read alignments with insertions/deletions were identified and re-aligned using GATK v3.7 using default parameters with minor modifications (LODThresholdForCleaning=0.4 defaultBaseQualities=30).

Sequence variations, including single nucleotide polymorphisms (SNPs) and insertion/deletion polymorphisms (indels) were called by BCFtools v1.3.1 on pileups generated by SAMtools v1.3.1. ^4^ Homozygous SNP and indel variants were identified using GATK’s SelectVariants to retain only variants with no support for the CS reference allele at a series of read depth thresholds (1, 5, 10, 20, 30 and 40). BEDTools v2.26.0 intersect tool was used to identify intersects between gene annotation coordinate ranges and the identified variants. Homozygous variants were analysed by SNPeff v4.3i ^5^ to estimate their impact in the context of the CS RefSeq v1.0 High Confidence gene annotations, excluding intergenic regions (-no-intergenic). Using all identified homozygous variants, we recalled the reference to generate a “Sonmez genome sequence v1.0”. Where there was no coverage of the CS reference, we softmasked the Sonmez genome sequence. It should be noted that these softmasked bases could represent regions which are either deletions in Sonmez or insertions in CS.

Finally, the read pairs that remained unmapped following the two-step alignment approach were assembled *de novo* to uncover Sonmez-specific genomic contigs. k-mers of length 71 bp and occurring ≥ 9 times in the unmapped reads were extracted using KMC v3.0.1. ^6^ These extracted k-mers were assembled into contigs using merutensils v0.7.15 kextend command; contigs < 250 bp in length were filtered out. This assembly approach ensures that contig extension only occurs if there is an unambiguous 1 bp extension possible in the input k-mer data set. *Methylobacterium* are well documented, common contaminants of reagents used in Illumina sequencing. As such, contigs showing high sequence identity to one of several *Methylobacterium* genomes (NZ_CP006992.1, NC_010511.1, NZ_CP017640.1, CP001029.1, AP014813.1, AP014810.1) or phiX (NC_001422.1) were also filtered out. These *de novo* assembled sequences are referred as “Sonmez-specific contigs” hereafter.

## Results

In total, 13.3 Gbp (91.51%) of the 14.5 Gbp CS reference genome assembly were covered by Sonmez reads, with a mean depth of coverage of ≈50×, enabling an almost complete, first construction of the Sonmez genome. Additionally, sequences that are either unique to Sonmez ( *e.g.* introgressions) or significantly divergent compared to CS were used to build up a *de novo* assembly. This assembly totaled 1.05 Gbp in length, with the longest contig being 15,887 bp (N50=427 bp, N90=269 bp). An updated version (v5.3p01) of the TriAnnot pipeline ^7^ optimized for wheat was used to generate similarity-based and *ab initio* gene models and annotate repetitive elements on contigs that are longer than 10 kilobases. While the *de novo* assembly was highly fragmented, compared to the recalled Sonmez genome, we were still able to pick up 35 gene models, of which 11 were high-confidence ( *Extended data* ^8^).

We identified between 3.15 – 23.96 million variants, depending on the coverage threshold used, of which between 0.03 – 3.23% were indel variants ( *Extended data* ^9^^,^^10^). We found that 1.47 – 2.39% of all variants fell within the RefSeq v1.0 High Confidence gene annotations ( *Extended data* ^9^). Of these, approx. 40% fell within coding regions. Of the homozygous variants supported by ≥ 5 reads, we observed approximately one variant per 500 bp in the A and B genomes and approximately one variant per 4,000 bp in the D genome.

Here, we present the complete genome of the elite wheat variety Sonmez, notable for its performance under low-irrigation conditions. In the face of climatic extremes and other factors that challenge the food safety of upcoming generations, genome sequences of multiple genotypes, varieties and close relatives will not only help us understand complex traits, such as yield and stress responses, but also enable us to efficiently explore the genetic diversity within germplasms for favorable genotypes and/or traits for crop improvement through the use of molecular tools.

## Data availability

### Underlying data

Sonmez complete genome sequence v1.0 and *de novo* assembly are available from the dedicated URGI database.

### Extended data

Figshare: Sonmez_Extended_Data1, https://doi.org/10.6084/m9.figshare.16992337. ^8^

This project contains the following extended data:

- Extended_data1_Sonmez_TriAnnotAnalysis_v1.xlsx (Gene models and repeat annotations of Sonmez-specific contigs)

Figshare: Sonmez_Extended_Data2, https://doi.org/10.6084/m9.figshare.16992322.v3. ^9^

This project contains the following extended data:

- Extended_data2_Sonmez_vs_CS_variantsummary_v1.pdf (Summary information of sequence variants between Sonmez and CS)

Figshare: Sonmez_Extended_Data3, https://doi.org/10.6084/m9.figshare.16992388.v2. ^10^

This project contains the following extended data:

- Sonmez.alt_fasta.vcf. (Homozygous SNP/indel variants identified between Sonmez and CS)

Data are available under the terms of the Creative Commons Attribution 4.0 International license (CC-BY 4.0).
