# Supplementary material for: The complete genome sequence of elite bread wheat cultivar, “Sonmez”
Source: F1000Res. 2022 Jun 6;11:614. [Version 1] doi: 10.12688/f1000research.121637.1 (PMC9194515; doi:10.12688/f1000research.121637.1)
Supplement: Peer review history

# Peer review: Reviewer response for version 1

**DOI:** 10.5256/f1000research.133524.r140015

**Published:** 2022-06-24

**Authors:** Gabriel Doredo Perez

**Affiliations:** 1 Dep. Bioquímica y Biología Molecular, Campus Rabanales C6-1-E17, Campus de Excelencia Internacional Agroalimentario (ceiA3), Universidad de Córdoba, Córdoba, Spain

The manuscript “The complete genome sequence of elite bread wheat cultivar, “Sonmez””, published on F1000Research by Akpinar et al, is an interesting work on structural genomics, covering both re-sequencing of 21 chromosomes of the A, B and D genomes (13.3 Gbp), as well as de novo sequencing (1.05 Gbp). Such latter interesting result represents either Sonmez-specific sequences, or sequences that considerably diverged between Sonmez and Chinese Spring (used as reference genome). The de novo assembly identified 35 gene models, of which 11 were annotated with high-confidence, that may contribute to the favorable traits of this variety. A total of 24 million sequence variants were identified, of which up to 2.4% reside in coding sequences.

 Interestingly, the Sonmez cultivar is resilient against certain biotic stressors, particularly soil-borne pathogens. Besides, it is notable for its performance under low-irrigation conditions. Therefore, this work is particularly relevant in the current trend of global warming and climate change, including worldwide drought. Comparison of wheat genomes will allow to decipher complex traits, like abiotic and biotic stresses. That should allow the development of molecular markers for wheat breeding. These developments will help to address future food demand for a growing worldwide population.

Are the datasets clearly presented in a usable and accessible format, and the assembly and annotation available in an appropriate subject-specific repository?

Yes

Are sufficient details of the sequencing and extraction, software used, and materials provided to allow replication by others?

Yes

Are the rationale for sequencing the genome and the species significance clearly described?

Yes

Are the protocols appropriate and is the work technically sound?

Yes

Reviewer Expertise:

Molecular biology.

I confirm that I have read this submission and believe that I have an appropriate level of expertise to confirm that it is of an acceptable scientific standard.

# Peer review: Reviewer response for version 1

**DOI:** 10.5256/f1000research.133524.r140013

**Published:** 2022-06-13

**Authors:** Zahide Neslihan Öztürk Gökçe

**Affiliations:** 1 Ayhan Şahenk Faculty of Agricultural Sciences and Technologies, Department of Agricultural Genetic Engineering, Niğde Ömer Halisdemir University, Niğde, Turkey

In the article, the authors performed reference-guided whole genome sequencing of an elite wheat cultivar Sonmez having high yield under stress conditions and specified with resistance against some biotic factors. The data are well presented and the information provided will be of great use for scientific community in the development of abiotic stress resilient wheat cultivars.

 Therefore my recommendation is to be accepted for indexing as it is. 

 Best regards

Are the datasets clearly presented in a usable and accessible format, and the assembly and annotation available in an appropriate subject-specific repository?

Yes

Are sufficient details of the sequencing and extraction, software used, and materials provided to allow replication by others?

Yes

Are the rationale for sequencing the genome and the species significance clearly described?

Yes

Are the protocols appropriate and is the work technically sound?

Yes

Reviewer Expertise:

Transcriptomics of abiotic stress tolerance

I confirm that I have read this submission and believe that I have an appropriate level of expertise to confirm that it is of an acceptable scientific standard.

# Peer review: Reviewer response for version 1

**DOI:** 10.5256/f1000research.133524.r140014

**Published:** 2022-06-13

**Authors:** Søren K. Rasmussen

**Affiliations:** 1 Department of Plant and Environmental Sciences, University of Copenhagen, Frederiksberg C, Denmark

A draft genome sequence of the hexaploidy wheat ‘Sonmez’, a Turkish wheat bread cultivar, is presented. A large number of sequence variants are identified, and as expected the highest density of these putative SNP markers are located on the A- and B-genome and much lower number on the D-genome. This can facilitate efficient markers-assisted selection taking advantage of Sonmez drought tolerance as emphasized.

Are the datasets clearly presented in a usable and accessible format, and the assembly and annotation available in an appropriate subject-specific repository?

Yes

Are sufficient details of the sequencing and extraction, software used, and materials provided to allow replication by others?

Yes

Are the rationale for sequencing the genome and the species significance clearly described?

Yes

Are the protocols appropriate and is the work technically sound?

Yes

Reviewer Expertise:

Molecular plant breeding, quality traits, plant genetic resources, grain cereals and legumes,

I confirm that I have read this submission and believe that I have an appropriate level of expertise to confirm that it is of an acceptable scientific standard.
